# Cytoplasmic Injection of Zygotes to Genome Edit Naturally Occurring Sequence Variants Into Bovine Embryos

**DOI:** 10.3389/fgene.2022.925913

**Published:** 2022-07-11

**Authors:** Jingwei Wei, Brigid Brophy, Sally-Ann Cole, Jannis Moormann, Jens Boch, Gӧtz Laible

**Affiliations:** ^1^ Animal Biotechnology, Ruakura Research Centre, AgResearch Ltd, Hamilton, New Zealand; ^2^ Institute of Plant Genetics, Leibniz Universität Hannover, Hannover, Germany; ^3^ Department of Molecular Medicine and Pathology, School of Medical Sciences, University of Auckland, Auckland, New Zealand

**Keywords:** Cas9, TALEN, genome editing, homology-directed repair, PMEL, PRLR, slick, cattle

## Abstract

Genome editing provides opportunities to improve current cattle breeding strategies through targeted introduction of natural sequence variants, accelerating genetic gain. This can be achieved by harnessing homology-directed repair mechanisms following editor-induced cleavage of the genome in the presence of a repair template. Introducing the genome editors into zygotes and editing in embryos has the advantage of uncompromised development into live animals and alignment with contemporary embryo-based improvement practices. In our study, we investigated the potential to introduce sequence variants, known from the pre-melanosomal protein 17 (*PMEL*) and prolactin receptor (*PRLR*) genes, and produce non-mosaic, edited embryos, completely converted into the precision genotype. Injection of gRNA/Cas9 editors into bovine zygotes to introduce a 3 bp deletion variant into the *PMEL* gene produced up to 11% fully converted embryos. The conversion rate was increased to up to 48% with the use of TALEN but only when delivered by plasmid. Testing three gRNA/Cas9 editors in the context of several known *PRLR* sequence variants, different repair template designs and delivery as DNA, RNA or ribonucleoprotein achieved full conversion rates up to 8%. Furthermore, we developed a biopsy-based screening strategy for non-mosaic embryos which has the potential for exclusively producing non-mosaic animals with intended precision edits.

## 1 Introduction

Selective breeding has a long and proven history for the incremental improvement of livestock. It is, however, curtailed by a lack of control of how genetic variants are re-distributed from parents to offspring by breeding, essentially preventing the desirable assembly of most or all beneficial allelic variants in individual animals. Instead, selection is used to pick animals with the best combinations of desirable vs undesirable variants. The invention of genome editing offers the possibility of augmenting conventional breeding approaches by directly introducing beneficial (or eliminating undesirable) sequence variants, even when only existing outside the breeding population, essentially within a single generation (Jenko et al., 2015; McLean et al., 2020; Mueller et al., 2021). Genome editing is based on programmable nucleases, including zinc finger nucleases, transcription activator-like effector nucleases (TALENs) and the RNA guided clustered, regularly interspaced, short palindromic repeat (CRISPR)/CRISPR-associated (Cas) nucleases (gRNA/Cas9), which can introduce unique, site-specific double-strand breaks into the genome (Chandrasegaran and Carroll, 2016). When supplied with a homologous donor repair template the cellular machinery can repair the damaged site by a homology-directed repair (HDR) mechanism, enabling the introgression of defined sequence changes. There are two principal editing approaches, i) editing the genomes of cells, which are then used to generate an animal, or ii) direct editing in one-cell embryos (Laible, 2018). Cell-mediated genome editing has the advantage of being able to fully characterize the edited genome prior to generating live animals. For livestock species, cell-mediated genome editing is reliant on primary cells and somatic cell nuclear transfer (SCNT) to generate edited animals from these cells (Tan et al., 2016). However, SCNT is associated with nuclear reprogramming inefficiencies leading to developmental problems that cause animal welfare concerns and result in low efficiencies for producing edited animals (Oback, 2008). The alternative comprises the delivery of editors into *in vitro* produced zygotes which has minimal impact on the embryo’s developmental potential and, by contrast, offers better live animal production efficiencies. Though, the embryo-mediated approach comes with its own drawback, which is the lack of control over timing and extent of editing in the embryo following the injection of editors. This can result in the generation of unintended and mosaic genotypes, rather than the precise and complete conversion of the embryo genome to produce animals with precisely edited homogenous genotypes for the rapid, targeted improvement of livestock (Mehravar et al., 2019). The main two delivery options for editors into zygotes are by electroporation and cytoplasmic microinjection. Electroporation is technically less demanding and enables simultaneous delivery into multiple embryos and has been efficiently used to generate knockout animals through the error prone non-homologous end joining (NHEJ) repair of the double-strand break (Lin and Van Eenennaam, 2021). However, using an additional homologous repair template to introduce precise HDR edits is difficult and more readily achievable through delivery by microinjection (Lillico et al., 2016; Niu et al., 2018; Wei et al., 2018; Zhou et al., 2018; Eaton et al., 2019; Park et al., 2020).

In our study, we focused on cytoplasmic injection of editors and donor templates into bovine zygotes with the aim to evaluate the ability to fully convert the genome into precisely edited, non-mosaic genotypes with this approach. As editing targets, we used naturally occurring sequence variants in two genes which are expected to provide improved thermotolerance. This included a 3 bp deletion in the signal peptide of the pre-melanosomal protein 17 (*PMEL*) that was shown to be the causative mutation for lightening the black coat colorings in Holstein-Friesian cattle (Laible et al., 2021). Lightening the coat color will reduce the radiative heat gain from sunlight which impacts on heat stress (Hansen, 1990). The other sequence variants comprised single base pair changes in the prolactin receptor (*PRLR*) gene known from tropical cattle, all generating pre-mature stop codons associated with the slick coat phenotype and improved thermotolerance (Littlejohn et al., 2014; Porto-Neto et al., 2018; Flórez Murillo et al., 2021). These editing targets were then used to determine the impact of TALEN and gRNA/Cas9 editors, the time of injection, donor templates and delivery of the editors as DNA, RNA or as ribonucleoprotein (RNP) complexes (for gRNA/Cas9 editors) on the conversion efficiency into completely edited genotypes.

In addition, we developed a screening strategy using embryo biopsies to gauge editing success in vitro produced embryos to enable the selective transfer of embryos that had been validated for the precisely edited genotype.

## 2 Materials and Methods

### 2.1 Genome Editors and Donor Repair Templates

Target-specific gRNAs were designed and cloned into the gRNA/Cas9 expression vector pX330 as previously described (Laible et al., 2021).

The repeat array assembly for the *PMEL*-specific binding sites of the TALENs was performed using the Golden TAL cloning method (Geiβler et al., 2011) with human codon-optimized parts and comprised the repeat variable diresidues (RVDs) HD-HD-NI-NG-NN-NG-NN-NN-HD-NG-HD-NG-NN-NI-NG-NN-NN-NN (TL17) and NG-HD-NG-NN-NG-NN-NN-NG-HD-HD-HD-NG-NI-HD-NI-NN-HD (TR217). The nuclease domain was used as a heterodimeric ‘sharkey’ FokI domain (TL17: DS variant; TR217: RR variant) (Guo et al., 2010). All TALENs had an HA epitope tag and a SV40 nuclear localization signal at their N-termini. Details about the TALENs and their target sites is shown in Supplementary Figure S1. The TALENs were inserted into a GoldenGate-compatible derivative of the vector pcDNA3 with a T7 promoter for *in vitro* transcription of TALEN mRNA and a CMV promoter driving the expression of the TALENs in embryos.

Plasmids encoding editors for transfection or injection were prepared with the PureLink™ HiPure Plasmid Midiprep Kit from Invitrogen (Invitrogen by Thermo Fisher Scientific). Synthetic gRNAs (Supplementary Table S1) were ordered from Synthego and Cas9 mRNA (GeneArt CRISPR Nuclease mRNA) and recombinant Cas9 (TrueCut™ Cas9 Protein v2) were bought from Thermo Fisher Scientific (Invitrogen by Thermo Fisher Scientific). TALEN mRNA was synthesized with Invitrogen’s mMESSAGE mMACHINE T7 Ultra Transcription Kit according to the manufacturer’s instructions, using XbaI-linearized TALEN plasmids as templates. The resulting capped, poly-A tailed *in vitro* produced mRNA was purified with MEGAclear™ Transcription Clean-Up Kit (Invitrogen by Thermo Fisher Scientific).

All single-stranded oligonucleotide (ssODNs) donor templates (Supplementary Table S2) were synthesized by Integrated DNA Technologies, Inc.

### 2.2 TALEN *in vitro* Cleavage Assay

TALENs were expressed using the TnT T7 Quick Coupled Transcription/Translation System (Promega) following the manufacturer’s instructions. 250 ng of each TALEN construct were used. The target DNA regions including TALEN-binding sites and either the wt or the three bp deletion allele were cloned via annealed oligos into pUC57. For the *in vitro* cleavage assay 4 µl of TnT reaction containing the TALEN pair proteins was mixed with 180 ng PvuI-linearized target DNA in 1 x NEBuffer 3 (New England Biolabs) supplemented with 2.5 µg/µl BSA in a total volume of 20 µl. After incubation for 60 min at 37°C, the reaction was inactivated at 65°C for 20 min and centrifuged at 16,000 g for 3 min. The supernatant (16 µl) was analyzed on a 1% agarose gel (Supplementary Figure S1).

### 2.3 *In vitro* Fertilization, Cytoplasmic Zygote Injection and Embryo Culture

*In vitro* fertilization (IVF) of *in vitro* matured oocytes aspirated from abattoir-derived ovaries and cytoplasmic zygote injection was carried out essentially as described (Wei et al., 2015). IVF zygotes were injected within 5h and 9h post fertilization unless at specific timepoints, as indicated in the main text. Zygotes were then co-cultured in groups of 10 embryos until the blastocyst stage at embryonic day seven.

Expression plasmids for gRNA/Cas9 and TALENs were injected at 20 ng/µl; mRNA for TALENs at 100 ng/µl and for Cas9 at 10 ng/µl (in combination with gRNA 129F, ssODN 1288), 10–20 ng/µl (in combination with gRNA 634, ssODN 1446), 20–75 ng/µl (in combination with gRNA 632, ssODN 1403); gRNA at 2 pmol/µl; Cas9 protein at 20 ng/µl; ssODNs at 100–200 ng/µl. The variations in concentrations were introduced as adjustment for improving embryonic development rates with specific editor, gRNA, repair template combinations.

### 2.4 Blastocyst Biopsy, Cryopreservation and Warming of Biopsied Blastocysts

The procedures were performed as described in detail (Wei et al., 2018). Briefly, 10–15 cells from the trophoblast opposite the inner cell mass were cut off with an ultra-sharp splitting blade (AB Technologies, NSW, Australia) under 200x magnification. Fetal calf serum was gradually added to the cutting drop to release the cell material from its adherence to the plastic surface of the culture dish. The biopsy was transferred in less than 1 µl to a PCR tube with 2.5 µl in phosphate-buffered saline (PBS) plus PVA and frozen at -20°C prior to subsequent lysis and genotyping.

The biopsied embryos were cryopreserved According to the CryoLogic Vitrification Method (CVM; cryologic.com/Cvm) Followed by Storage in Liquid Nitrogen

To recover embryos from cryopreservation, vitrified embryos were removed from liquid nitrogen and warmed by sequentially immersing them in Embryo Hold medium with decreasing concentrations of sucrose for 2 min (0.27 M sucrose), 3 min (0.16 M sucrose) and 2.5 min (no sucrose), respectively, at 38.5°C. Thereafter embryos were incubated in fresh Embryo Hold medium for 2–3 h and morphologically assessed.

### 2.5 Transfection of Primary Bovine Cells

Culture and transfection of bovine fetal fibroblast has been described (Laible et al., 2021). Briefly, the expression plasmids for gRNA/Cas9 editors (1 µg) were transfected into 2 × 10^5^ primary bovine fetal fibroblast cells per sample. Transfections were done in duplicate using a 10 µl tip with program C4 (1400 V pulse voltage, 20 msec pulse width, 2 pulse) according to the manufacturer’s instruction of the Neon transfection system (Invitrogen). Following *in vitro* culture for 48 h, the transfected cells were harvested, lysed in 20 µl protein K (0.2 µg/µl) containing PCR buffer for 30 min at 50°C followed by deactivation of the protein K at 95°C for 15 min. The lysate (2 µl) was directly used for droplet digital PCR (ddPCR) analysis.

### 2.6 Analysis of Cells and Embryos for HDR Editing by ddPCR

Prior to PCR, blastocysts were lysed as described above for transfected primary bovine fetal fibroblasts, except for using a 10 µl reaction volume. The relevant target regions were pre-amplified by 13 cycles from 2 µl of the embryo lysate using a Kapa 2G Fast Hotstart PCR kit (KapaBiosystems) in a in 10 µl reaction with 0.5 pmol/µl of each primer using the following amplification cycle: 15 s at 95°C, 15 s at 60°C, 1 s at 72°C and a pre- and post-cycle step of 3 min at 95 and 72°C for 5 min, respectively. Embryo biopsies were lysed in 10 µl 1 µg/µl BSA (Roche) for 15 min at 95°C. Using the same conditions as above, the target regions were pre-amplified for 17 cycles.

Cell lysate (2 μ1), pre-amplified embryo (2 μ1) and biopsy reactions (2 μ1) were used as template in ddPCR assays. The ddPCR assays were performed on a QX200 Droplet Digital PCR System (Bio-Rad) essentially as described (Laible et al., 2021). All reactions included a FAM-labelled HDR probe, a HEX-labelled reference probe and an unlabelled dark probe. The sequences of probes used in the assays have been summarized in Supplementary Table S3. Cycle conditions were 95°C for 10 min, followed by 40 cycles of 94°C 30 s, 60°C (for *PMEL*) or 57°C (for *PRLR* 632) or 59°C (for *PRLR* 631 and 634) 1 min (ramp rate 2°C/s), followed by a final 10 min at 98°C. Results were evaluated with a Bio-Rad Droplet Reader and the QuantaSoft™ Analysis Pro Software (Bio-Rad). All PCR primers are listed in Supplementary Table S4.

### 2.7 Statistical Analysis

Statistical significance levels of observed differences were determined by two-tailed t-tests.

## 3 Results

### 3.1 Editors for the Introduction of Naturally Occurring Sequence Variants

**FIGURE 1:**
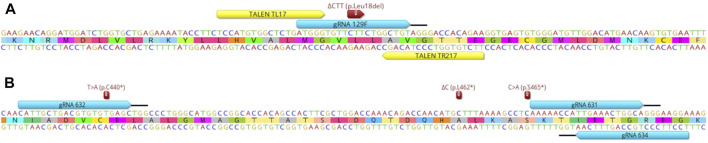
Overview of editor binding sites and relevant naturally occurring mutations relative to the target region within the *PMEL* and *PRLR* genes. **(A)** Shown is the DNA and amino acid sequence of the *PMEL* target region with the location of the ΔCTT (*p*.Leu18del) mutation. Binding sites of gRNA/Cas9 129F and TALEN pair TL17/TR217 are indicated by the blue and yellow boxes, respectively. The black bar indicates the protospacer adjacent motif (PAM) sequence of the gRNA/Cas9 editor. **(B)** Equivalent information for the location of three *PRLR* mutations T > A (*p*.C440*), ΔC (*p*.L462*), C > A (*p*.S465*) and binding and PAM sites of the gRNA/Cas9 editors 631, 632 and 634 in relation to the *PRLR* sequence.

We aimed at introducing natural sequence variants in the *PMEL* and *PRLR* genes thought to increase thermotolerance of cattle (Schmutz and Dreger, 2013; Littlejohn et al., 2014; Porto-Neto et al., 2018; Flórez Murillo et al., 2021; Laible et al., 2021). To edit the *p*.Leu18del PMEL mutation, caused by a 3bp deletion associated with coat color dilution, we tested gRNA 129F/Cas9, previously used to generate fully edited calves by cell-mediated HDR editing (Laible et al., 2021), and the TALEN pair TL17/TR217 (Figure 1A). Multiple *PRLR* sequence variants were described associated with the thermotolerant slick hair phenotype (Littlejohn et al., 2014; Porto-Neto et al., 2018). In our study, we evaluated zygote-mediated HDR editing of three PRLR mutations, *p*.C440*, *p*.L462* and *p*.S465*, resulting from a T to A (T > A), deletion of a C (ΔC) and C to A (C > A) base change, respectively with gRNA/Cas9 editors (Figure 1B). A comparison of the sequences of the wild type (wt) allele and these mutant alleles with corresponding changes to the reading frames is depicted in Supplementary Figure S1.

### 3.2 Editing Activity in Embryos

**FIGURE 2:**
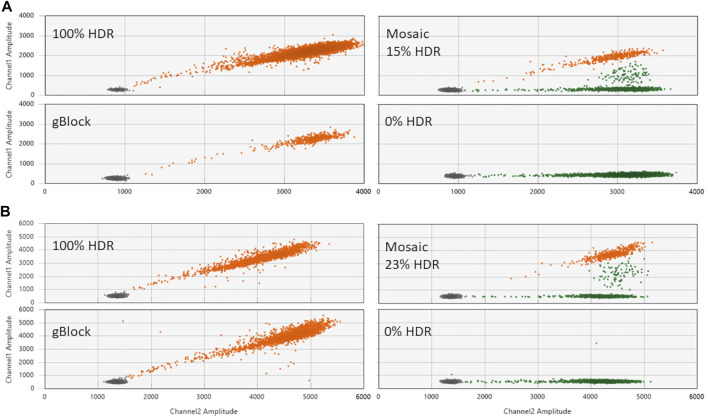
Evaluation of the contribution of intended *PMEL* and *PRLR* mutations in edited embryos by ddPCR. **(A)** Shown are 2D amplification plots generated with three different embryos that were injected with plasmid encoded TALENs TL17/TR217 plus ssODN 1288 and analyzed with the HDR probe 1321 for the PMEL ΔCTT (*p*.Leu18del) mutation. The panel labeled as gBlock depicts the assay results for a synthetic DNA fragment (1282) comprising the PMEL ΔCTT (*p*.Leu18del) mutation and represents a positive control for 100% HDR. **(B)** ddPCR results for three different embryos injected with plasmid encoded gRNA 632/Cas9 plus ssODN 1403 and analyzed with the HDR probe 1554 for the *PRLR* T > A (*p*.C440*) mutation. The 100% HDR control panel (gBlock) was done with synthetic DNA fragment 1413. Orange dots represent HDR-positive droplets, green droplets represent wt and non-HDR indel alleles and black dots represent droplets with no amplification. Contributions of the intended HDR alleles are given for each plot with examples for embryos that were fully converted to the HDR genotype (100% HDR), only partially HDR edited embryos (Mosaic) and embryos without edited HDR alleles (0% HDR). Channel1 Amplitude: FAM signal (HDR probe); Channel2 Amplitude: HEX signal (reference probe).

To confirm HDR editing activity, plasmid-encoded editors plus a ssODN specifying the intended HDR edit were injected into bovine zygotes. After culture to the blastocyst stage, embryos were analyzed for the intended HDR edits by ddPCR. Representative ddPCR results for the three principal categories of embryos generated by zygote-mediated HDR editing are shown in Figure 2. Embryos can either be fully converted to the HDR genotype (100% HDR), partially HDR edited (Mosaic) or without HDR edited alleles (0% HDR). The latter category could be comprised of unedited wt alleles, unspecified indel alleles as a result of NHEJ repair or a mixture thereof. The genotypes of these embryos were not further analyzed as they represented undesirable genotypes.

For the *PMEL* mutation, injection of the gRNA/Cas9 editor 129F with ssODN 1288, produced 86% of embryos that were positive for the HDR edit (Table 1). However, most showed various degrees of mosaicism and only 9% were identified as fully converted into the HDR genotype.

**TABLE 1:** HDR editing efficiencies in bovine embryos for *PMEL* and *PRLR* mutations with different editors.

Editor	ssODN	Target Mutation	PAM Mutation	No. of Blastocysts	HDR + ve	100% HDR
gRNA 129F/Cas9	1288	*PMEL*: ΔCTT (*p*.Leu18del)	No	64	55/64 (86%) ^a^	6/64 (9%) ^a^
TALEN TL17/TR217	1288	*PMEL*: ΔCTT (*p*.Leu18del)	NA	66	68/69 (99%) ^b^	33/69 (48%) ^b^
gRNA 632/Cas9	1403	*PRLR*: T > A (*p*.C440*)	Yes	49	22/49 (45%) ^a^	3/49 (6%) ^a^
gRNA 634/Cas9	1446	*PRLR*: ΔC (*p*.L462*)	Yes	25	7/25 (28%) ^a^	1/25 (4%) ^ab^
gRNA 634/Cas9	1411	*PRLR*: ΔC, C > A (*p*.L462*, *p*.S465*)	Yes	151	96/151 (64%) ^b^	0/151 (0%) ^b^
gRNA 631/Cas9	1344	*PRLR*: ΔC (*p*.L462*)	Yes	7	4/7 (57%) ^ab^	0/7 (0%) ^ab^

^ab^Values for *PMEL*, and *PRLR*, editing with different superscripts within a column differ *p* < 0.01.

With a three base pair deletion there was also the possibility to design TALENs that could discriminate between edited and wt alleles. To avoid functional binding and recleavage of the edited, three bp deletion allele, we tested two alternative short regions (17 aa, 28 aa) between the repeat and FokI domains of the TALEN to limit their activity to a distinct spacer size between the two TALEN binding sites. In addition, we designed different combinations of TALEN to bind with a small spacer (12 or 10 bp) for the target wt allele, such that the spacer in the edited allele (reduced to only 9 or 7 bp) would be too short for TALEN activity. To test which TALEN configuration is suitable to discriminate between edited and wt allele, the TALEN activities were first determined via *in vitro* cleavage assays (Supplementary Figure S2). TALEN combinations that bind with the shorter spacer (10 bp) in the wt allele did not show any residual activity on the edited allele (7 bp spacer) and we chose the TALEN pair TL17/TR217 for further experiments.

HDR editing could be detected in 99% of blastocysts derived from embryos injected with the TALEN pair plus ssODN 1288. Almost half of the embryos (48%) were non-mosaic and fully converted into the intended HDR genotype (Table 1). This showed that the TALEN pair was the more efficient editor for the introduction of the three bp deletion compared to gRNA 129F/Cas9.

All three *PRLR* mutations associated with the slick coat phenotype were single base pair changes (Littlejohn et al., 2014; Porto-Neto et al., 2018) and for gRNA/Cas9 editors 631 and 634 the intended sequence change was also outside of their target-specific binding site (Figure 1B). To prevent recleavage of the edited allele, we therefore included a protospacer adjacent motif (PAM) blocking mutation in addition to the PRLR mutations in the ssODN sequence for improved editing efficiencies. All gRNA/Cas9 ssODN donor combinations were able to generate embryos comprising alleles with the intended ΔC (*p*.L462*) HDR edit with efficiencies up to 64% (Table 1). The highest proportion was obtained with donor 1411 which included the additional C > A (*p*.L462*, *p*.S465*) slick mutation closer to the gRNA 634/Cas9 cleavage site (Figure 1B) which might have boosted the overall editing outcome. However, all edited embryos were mosaic with none of the embryos fully converted (100% HDR). Complete conversion into the HDR edited genotype was only observed with two combinations at 6% (gRNA 632/Cas9; ssODN 1403) and 4% (gRNA 634/Cas9; ssODN 1446) of the injected embryos.

### 3.3 Donors

For the introduction of the PMEL *p*.Leu18del mutation, we used the previously validated ssODN donor 1288 (Laible et al., 2021). This donor was comprised of target strand sequence (opposite strand to PAM sequence) and did not include a PAM blocking mutation. Alternative ssODN donors for the PMEL mutation were not evaluated.

With relatively low editing efficiencies into fully converted slick genotypes, we tested different ssODN donors in conjunction with the *PRLR* gRNA/Cas9 editors 632 and 634 for their HDR potential in transfected primary bovine fibroblasts (Table 2). Following co-transfection of editors and ssODN donors, transfected cell pools were analysed for the presence of the edited T > A (*p*.C440*) or ΔC (*p*.L462*) allelic variants.

**TABLE 2:** HDR editing efficiency of different gRNA/Cas9 editor and donor combinations in transfected primary bovine cells.

Editor	ssODN	Strand Sequence	Length (bp)	Homology Arm Left/Right (bp)	PAM Mutation	% HDR PRLR:	% HDR PRLR:
						T > A (*p*.C440*)	ΔC (*p*.L462*)
632	1403	non-target	87	40/46	yes	3	NA
	1404	Target	87	40/46	yes	6	NA
	1538	non-target	82	40/41	no	4	NA
	1539	Target	82	40/41	no	6	NA
634	1411	Target	104	40/51	yes	NA	5
	1412	non-target	104	40/51	yes	NA	0
	1536	target	110	40/57	no	NA	3
	1537	non-target	110	40/57	no	NA	1
	1446	target	104	40/63	yes	NA	5
	1534	target	110	40/69	no	NA	2
	1535	non-target	110	40/69	no	NA	1

Based on the embryo editing results, we started with donor 1403, comprising non-target strand sequence (PAM sequence containing strand) for editing the T > A (*p*.C440*) mutation with gRNA 632/Cas9. Cell pools of co-transfected cells analyzed by ddPCR showed a contribution of 3% for the HDR genotype. Using the donor ssODN 1404 targeting the same sequence but on the opposite (target) strand, increased the contribution of the HDR genotype to 6%. A slightly shorter pair of ssODN (1538, 1539) with target and non-target strand sequences gave a similar result despite these donors not including a PAM blocking mutation. The target strand sequence ssODN 1539 was correlated with a higher proportion of the HDR genotype (6%) than the non-target strand sequence donor 1538 (4%).

For introducing ΔC (*p*.L462*) in conjunction with gRNA 634/Cas9, ssODN donor 1411, which has a target strand sequence and produced 64% of injected embryos with ΔC (*p*.L462*) edits (Table 1), was associated with a 5% proportion for the ΔC (*p*.L462*) edited PRLR sequence in co-transfected cell pools. The complementary ssODN donor 1412 failed to introduce the ΔC (*p*.L462*) mutation which could not be detected (0%). For the equivalent target strand donor to 1411 but without the PAM blocking mutation (1536), the HDR edited proportion in co-transfected cell pools was with 3% lower than with an additional PAM mutation. In contrast to donor 1412, the non-target strand sequence ssODN without PAM mutation (1537) showed some activity (1%) for successful editing of the ΔC (*p*.L462*) mutation but lower compared to 1536.

The target strand sequence donor 1446, which in embryos was associated with a 4% rate for full conversion, was compared to a pair of ssODN target/non target strand sequence donors without a PAM blocking mutation. In cells, the use of the ssODN donor 1446 resulted in an overall contribution of 5% of ΔC (*p*.L462*) alleles. This was comparable to 1411 suggesting that the inclusion of the second slick mutation (C > A, *p*.S465*) on this donor did not increase the incorporation of the ΔC (*p*.L462*) edit. The target/non-target-strand equivalents to 1446 without PAM mutation showed a reduced editing rate compared to 1446 (5% vs 2% and 1%). Again, the target strand sequence donor (1536) was the better performing ssODN (2% vs 1%).

### 3.4 Injection Time

After injecting into a zygote, editing would ideally start and be completed at the one-cell stage. However, once editors are injected, there is no control over the events that take place in the embryo, which is often causing the production of mosaic embryos. Hence, the time of injection is a critical parameter for delivering editors during early stages permissive for HDR editing. HDR activity peaks during the G1/S-phase of the cell cycle (Mao et al., 2008; Heyer et al., 2010) with S-phase of the first zygotic cell cycle in bovine embryos beginning approximately 9h post IVF (Comizzoli et al., 2000). To best align the presence of functional editors with the window of peak HDR activity, we tested a range of different zygote injection times. Using plasmid-encoded gRNA 634/Cas9 together with ssODN 1411, we have evaluated the impact of injection time on HDR outcomes for 1 hour time windows between 5 h and 9 h post IVF (Table 3). Embryos injected within different periods were co-cultured in separate groups and assessed for HDR editing by ddPCR at the blastocyst stage. The percentage of embryos with HDR edits ranged from 49% at 5–6 h post IVF to 62% at 7–8 h post IVF (Table 3). However, the observed variations of percentages across the entire time window were relatively small and not significantly different. There was also no difference in efficiency of producing fully converted (100% HDR) embryos. We therefore injected larger batches of embryos in further experiments within the 5h–9h window without separating embryos into groups of hourly injection times.

**TABLE 3:** Effect of time of injection on HDR editing with plasmid-encoded gRNA 634/Cas9 in bovine embryos.

Injection Time	Injection Sessions	No. Blastocysts	HDR + ve Embryos	100% HDR
5–6 h post IVF	3	33	16/33 (49%)	1/33 (3%)
6–7 h post IVF	3	36	22/36 (61%)	1/36 (3%)
7–8 h post IVF	2	26	16/26 (62%)	0/26 (0%)
8–9 h post IVF	3	17	10/17 (59%)	0/17 (0%)

### 3.5 DNA, RNA, RNP Delivery of Editors

Genome editors can be delivered by expression plasmids (DNA), as RNA (mRNA and gRNA) or as recombinant proteins and for gRNA/Cas9 as RNP complex. The choice of delivery has implications on when and for how long the editors can be active. With expression plasmids, there will be a delay as the editors need first to translocate into the nucleus, be transcribed and then translated in the embryo. mRNA only needs to be translated and the protein or ribonucleoprotein complexes are immediately active. Whether expressed from DNA or RNA or delivered as protein, impacts also on how long the editors remain functional in the embryo (Kim et al., 2014). The stability of plasmids results in prolonged editing activity, whereas RNA and proteins are more quickly turned over associated with a shorter window of activity. To evaluate which delivery option would produce the most non-mosaic embryos, fully converted into the HDR genotype, we compared injection of editors as DNA, RNA and RNPs. (Table 4).

**TABLE 4:** HDR outcomes in embryos dependent on editor delivery as DNA, RNA or RNP.

Mutation	Editor	Delivery	Mosaic HDR	Complete HDR
*PMEL*: ΔCTT (*p*.Leu18del)	gRNA 129F/Cas9, ssODN 1288	DNA	85% (60/71) ^a^	8% (6/71)
		RNA	60% (50/84) ^b^	11% (9/84)
		RNP	17% (8/47) ^c^	6% (3/47)
	TALEN TR17/TL217, ssODN 1288	DNA	57% (42/74) ^a^	39% (29/74) ^a^
		RNA	90% (63/70) ^b^	3% (2/70) ^b^
*PRLR*: ΔC (*p*.L462*)	gRNA 634/Cas9, ssODN 1446	DNA	18% (4/22)	5% (1/22)
		RNA	25% (6/24)	0% (0/24)
		RNP	42% (5/12)	8% (1/12)
*PRLR*: T > A (*p*.C440*)	gRNA 632/Cas9, ssODN 1403	DNA	39% (19/49) ^a^	6% (3/49)
		RNA	64% (48/84) ^b^	7% (6/84)

^abc^Values with different superscripts within a column differ *p* < 0.05.

Delivery of the gRNA 129F/Cas9 as plasmid (together with the donor 1288) produced a high number of mosaic embryos with HDR-edits (85%) which were significantly lower for injection with RNA (60%) and RNPs (17%). More important is the ability to efficiently support the conversion into non-mosaic, fully converted embryos. The best complete conversion was observed with RNA (11%) while plasmid and RNP delivery achieved 8% and 6%, respectively. However, these differences were not statistically significant.

Injection of the TALEN pair TL17/TR217 as mRNA with donor 1288 produced a high rate of mosaic embryos with HDR edits (90%) but low numbers of fully converted embryos (3%). In this case, plasmid delivery of the TALENs was much better compared to mRNA delivery, and 38% of embryos were genotyped as completely converted into the intended PMEL HDR-edited genotype. Protein delivery of the TALENs TR17 and TL217 could not be tested as they were not available as recombinant proteins for the study.

For editing of the *PRLR* ΔC (*p*.L462*) mutation with gRNA 634/Cas9 and ssODN donor 1446, generation of mosaic embryos with HDR edits tended to increase for deliveries as DNA (18%), RNA (25%) and RNP (42%). A similar trend did not extend to the efficiencies for complete conversion into the HDR genotype which were 5% for DNA, 0% for RNA and 8% for RNP delivery. In addition, we evaluated DNA and RNA delivery of the gRNA 632/Cas9 with donor 1403 for the introduction of the *PRLR* T > A (*p*.C440*) mutation. Here, conversion rates into fully HDR edited embryos was similar with 6% for DNA and 7% for RNA.

### 3.6 Determining HDR-Editing Outcomes With Embryo Biopsies

The use of mosaic bovine embryos to produce edited cattle is problematic because of the welfare cost for what are effectively unwanted animals and the immense efforts that would be required to subsequently breed non-mosaic edited cattle given the long generational interval and single offspring per generation. Screening of embryo biopsies might provide a strategy to identify fully edited embryos that would enable exclusive production of animals with the intended edited genotype. Therefore, we next investigated this approach by using the plasmid encoded TALEN pair TL17/TR217 with the donor 1288 for zygote injections. Biopsies of 10–15 cells representing approximately 10% of the embryo were taken at the blastocyst stage and analyzed for the degree of intended HDR editing by ddPCR (Table 5). We observed a small number of biopsies (2%) which lacked contributions from the ΔCTT HDR edit (0% HDR). Most biopsies (51%) showed a mosaic genotype but, a substantial proportion (47%) was non mosaic, fully converted into the ΔCTT *PMEL* genotype (100% HDR) which was consistent with what we had observed with the analysis of whole embryos (Tables 1 and 4). Biopsies were also screened from embryos injected with gRNA 632, Cas9 mRNA and ssODN donor 1403 for HDR editing the *PRLR* mutation T > A (*p*.C440*). Compared to the *PMEL* editing, a larger proportion of biopsies appeared to remain unedited for the intended sequence change (38%) while the number of mosaic biopsies (55%) was similar to what we determined for the editing of the *PMEL* mutation. As we had observed before, the efficiency for full conversion was much lower for the *PRLR* mutations. Only 7% of the biopsies displayed a full conversion into the intended HDR genotype.

**TABLE 5:** Editing status of embryos determined from biopsies.

Variant	Editor	Biopsies	0% HDR	Mosaic HDR	100% HDR
*PMEL*, ΔCTT	TALEN	43	1/43 (2%) ^a^	22/43 (51%)	20/43 (47%) ^a^
*PRLR*, T > A	gRNA/Cas9	87	33/87 (38%) ^b^	48/87 (55%)	6/87 (7%) ^b^

^ab^Values with different superscripts within a column differ with *p* < 0.01.

### 3.7 Impact of Manipulations on Embryonic Development and Survival

For practical reasons it will be necessary to cryopreserve biopsied embryos allowing screening of the biopsy material and accumulation of embryos fully validated for the intended edits. However, embryos can be very sensitive to manipulations which can negatively affect their developmental potential. Therefore, we wanted to investigate the impact of the cytoplasmic injection, biopsy and cryopreservation of embryos on their development and survival after cryopreservation. Injection resulted in a slightly reduced rate for the total development into blastocysts counting all blastocysts assessed as grade 1—3 (G1-3). Total development of non-injected embryos reached 50%, whereas for the injected embryos we observed development rates between 31% and 42% (Table 6). For the injected embryos, total development was relatively stable, irrespective of editor delivery as DNA or RNA and whether TALENs or gRNA/Cas9 editors were used. By contrast, development to grade 1–2 (G1-2) blastocysts, a suitable embryo quality for transfer into recipients for development to term, was not higher for non-injected embryos (13%) than for injected embryos (6%–22%). Only the injection of gRNA 632 with Cas9 mRNA showed a slightly lower development rate to G1-2 embryos (6%) compared to the other treatments (13%–22%).

**TABLE 6:** Embryo development and survival following biopsy and cryopreservation.

Target	Zygote Injection	No. Zygotes in IVC	No. G1-3 Blastocysts	No. G1-2 Blastocysts	No. Embryos Biopsied and Cryopreserved	No. Embryos Re-expanded
PMEL	TL17/TR217 DNA, 1288	356	148/356 (42%) ^a^	45/356 (13%) ^a^	45/356 (13%)	26/27 (96%)
PMEL	TL17/TR217 mRNA, 1288	98	30/98 (31%) ^a^	14/98 (14%) ^a^	14/98 (14%)	11/11 (100%)
PRLR	gRNA 632/Cas9 DNA,1403	72	31/72 (43%) ^a^	16/72 (22%) ^a^	16/72 (22%)	14/14 (100%)
PRLR	gRNA 632, Cas9 mRNA,1403	243	87/243 (36%) ^a^	15/243 (6%) ^b^	15/243 (6%)	6/6 (100%)
NA	Not injected	209	104/209 (50%) ^b^	28/209 (13%) ^a^	28/209 (13%)	24/24 (100%)

^ab^Values within different superscripts within a column differ with *p* < 0.05.

Survival following cryopreservation was determined by thawing the embryos and morphological assessment whether the thawed embryos fully re-expanded as indicator for their recovery from cryopreservation. All tested embryos, except one, recovered well from cryopreservation irrespective of whether they had been injected or not (96%–100%). It also showed that taking biopsies of embryos is not compromising their recovery from cryopreservation which suggests that injected, biopsied embryos can be cryopreserved with high efficiency for future transfer and development to term.

## 4 Discussion

The ability for the efficient introgression of beneficial sequence variants by genome editing has the potential for accelerating the improvement of livestock. Applications have so far mainly focused on editing primary cells and the use of SCNT to generate precisely edited livestock from these cells (Bishop and Van Eenennaam, 2020). While access to many cells makes it possible to isolate correctly edited cells even at relatively low HDR editing efficiencies, production of live animals by SCNT is fraught with inefficiencies causing low pregnancy rates and undesirable animal welfare issues. In our study, we evaluated the direct HDR editing of embryos, potentially allowing the targeted introgression into the latest genetics for minimal genetic lag. Few reports have described the use of zygote-mediated HDR editing in livestock. These demonstrated that HDR-editing is readily achievable but produced more animals with non-edited, indel and mosaic genotypes (Niu et al., 2018; Zhou et al., 2018; Eaton et al., 2019; Park et al., 2020). Our aim was to evaluate different parameters to optimise the conversion of injected zygotes into fully converted, non-mosaic embryos with the intended precisely edited genotype.

The editor/donor combination, gRNA 129F/Cas9; ssODN 1288, we had previously validated in primary bovine cells for the introduction of a 3bp *PMEL* deletion, generated a high percentage of embryos comprising HDR alleles. The majority were mosaic and only a small fraction (9%) was fully converted into the intended HDR genotype. Almost half of the injected embryos were fully converted with the use of the TALENs TL17/TR217 as editor. This TALEN pair was carefully designed to be selective for the non-edited wt allele and only able to introduce a double-strand break into the wt allele, while the shortened spacer region between the TALEN pair in the HDR-edited allele prevented it. Although we have not formally shown it, this can be assumed to be also true for the gRNA 129F/Cas9, where the three bp deletion represents a substantial change to the 20bp gRNA spacer region with homology to the target site. Hence, it was surprising that the TALEN in this study showed a much higher efficiency for a complete conversion of the target sequence in comparison to the gRNA/Cas9 editor.

Although both genome editing tools have in general been reported with comparable editing efficiencies, they exhibit particular differences how they find and access target sites in chromatin (Sternberg et al., 2014; Cuculis et al., 2016; Jain et al., 2021). TALENs have also a greater tolerance for alleles with small indel mutations of incorrectly edited alleles compared to gRNA/Cas9 editors with the potential for recleavage providing additional HDR opportunity, resulting in an overall higher replacement efficiency. Furthermore, it is important to note that the cleavage sites for gRNA 129F/Cas9 and TL17/TR217 are not identical (Figure 1). The target site includes a small repeat and only the TALENs cleave at the center of this duplication. Beside HDR, this double-strand break could also be a target for repair by microhomology-mediated end joining which, together with the other functional differences, might have contributed to the high conversion rates we observed with the TALENs (Iyer et al., 2019).

For the single base pair *PRLR* mutations, we limited our editing to gRNA/Cas9 editors which provide the option to introduce a silent blocking mutation in the PAM sequence to prevent recleavage of the HDR-edited allele. Still, only modest (<10%) full conversion efficiencies could be achieved. In part, this might have been caused by a lack of suitable PAM sites (at least for two of the gRNA/Cas9s) to position the editors closer to mutation target site for more efficient HDR (Elliott et al., 1998). The development of a series of Cas9 variants with relaxed PAM requirements or in its most recent iteration a ‘near PAMless’ Cas9 will reduce this limitation, albeit with a small trade-off on efficiency (Walton et al., 2020). Introduction of single base pair changes could also be achieved with base editors (Komor et al., 2016; Nishida et al., 2016; Gaudelli et al., 2017). Although positioning is equally crucial, they function without the need for double-strand breaks and essentially lack the potential for introducing unwanted indel mutations resulting from NHEJ repair. However, the available cytosine and adenine base editors can only introduce transition mutations and are incapable of editing the *PRLR* T > A and C > A base change transversion mutations. This limitation was abrogated with the development of the prime editing platform, which is based on a fusion of a Cas9 nickase with a reverse transcriptase domain used with an extended gRNA, termed pegRNA for prime editing guide RNA (Anzalone et al., 2019). The pegRNA combines the target-specific spacer sequence of a standard gRNA with a 3′ extension as a template for the reverse transcriptase encoding the desired edits which can be small insertions, deletions and all possible base pair changes. Initial problems that we and others experienced were most likely related to pegRNA instability issues, which were addressed by adding a structured RNA pseudoknot at the 3’ end to prevent degradation (Nelson et al., 2021). Recently, further improvements of prime editing efficiencies were achieved with the simultaneous inhibition of mismatch repair (Chen et al., 2021). Prime editors can also be paired for enhanced efficiency and greater range of precise sequence changes which holds much promise for the targeted genetic improvement of livestock (Anzalone et al., 2021; Choi et al., 2021; Lin et al., 2021).

With our interest in small sequence changes, we have focused on the use of ssODNs donors which have been validated as efficient repair templates. Asymmetry and donors of target strand sequence were reported to enhance HDR (Richardson et al., 2016). In our own experience, asymmetry had little effect (unpublished observation), whilst strand preference was less clear. A study using a human cell model observed a Cas9 preference for ssODNs of the target strand and Cas12a (Cpf1) for the non-target strand (Wang et al., 2018a). HDR editing with Cas9 in bovine embryos appeared to show a strong preference for the non-target strand (Park et al., 2020). We therefore evaluated the impact of using donors with target and non-target strand sequences in primary bovine cells. Target and non-target strand ssODNs facilitated the introduction of template-specified mutations, with one notable exception (Table 2). The non-target strand ssODN 1412 showed no detectable HDR-editing activity. Overall, the target strand ssODNs tended to be better compared to the non-target strand ssODNs. We also included some donors that lacked a PAM blocking mutation and can generate edited alleles that remain targets for recleavage by Cas9. All had the potential for HDR editing in bovine cells, albeit in combination with gRNA634/Cas9 at relatively low levels. Whether they would be suitable for generating fully converted embryos remains to be seen but might provide an option when the simultaneous introduction of a PAM blocking mutation is unwanted.

The time of injections of editors into livestock zygotes varies widely between different studies ranging from 0 to 24 h post IVF [summarized in (Hennig et al., 2020)]. Injection into oocytes prior to IVF (0 h) might lead to differential editing of the paternal and maternal genomes (Suzuki et al., 2014). Following fertilization, the paternal genome will first decondense providing access of editors, while the maternal genome is still in a condensed state preventing access, which could result in incomplete editing. In bovine, oocyte injection reduced the rate of mosaicism by 70–90% when compared to injections performed 20 h post IVF irrespective of editor delivery as RNA or RNP. However, the same level of reduction was observed with injections at 10 h post IVF (Lamas-Toranzo et al., 2019). A reduction of mosaicism in pig embryos was also reported for injection of *in vivo* derived oocytes prior to fertilization when compared to injection times of 5h–6h post fertilization (Navarro-Serna et al., 2021). However, the reduction in mosaicism was only achieved with delivery by RNPs, but not by RNA. For livestock, there have been few studies that reported on precision editing by HDR following injection into zygotes generated following insemination or IVF with examples for sheep, goat, cattle and pig (Wei et al., 2015; Lillico et al., 2016; Niu et al., 2018; Wei et al., 2018; Zhou et al., 2018; Eaton et al., 2019; Park et al., 2020). All have reported the ability of the approach for full or close to full conversion into the HDR genotypes. Although the time of injection relative to fertilization was not provided for all studies, full conversion into the precisely edited genotypes was achievable with injections of the editing tools at 6 h, 8 h and 18 h post IVF for bovine embryos. In the present study, we attempted to further optimize the injection time as our previous work had suggested that injection at 8 h post IVF has lower rates of mosaicism compared to 18 h (Wei et al., 2015). Focusing solely on full conversion rates, there was no significant difference between injections from 5 h to 9 h post IVF. Possibly this is already an excellent time window for the injection and other factors might have greater impact in increasing full conversion rates.

With the aim to fully convert the genotype following the injection of zygotes, one needs to strike a balance between immediate onset of editing at the 1 cell stage and editors to remain active long enough to ensure complete conversion of all alleles. This differs depending on the delivery of editors as DNA, RNA or RNP (Wei et al., 2015; Glass et al., 2018). All delivery options have been validated to enable the generate HDR-edited livestock, although to various degrees of efficiencies (Bishop and Van Eenennaam, 2020; Park et al., 2020). Plasmids are inexpensive and stable molecules that provide a robust delivery option. However, they carry the risk that they can become integrated at random off-target sites or in combination with editor-mediated double-strand breaks, at on-target sites (Graham et al., 2009; Kim et al., 2014; Young et al., 2020). Still, integration of a circular plasmid is a rare event and animals can be readily tested for integrated plasmids by endpoint PCR, or sequencing. In this study, we have not implemented PCR testing for plasmid integration because we occasionally observed low level amplification products from biopsies. This made it too unreliable and might have been caused by lingering plasmid fragments still present at low concentrations in the embryo biopsy. The problem is absent when using the alternative delivery options RNA and RNP that provide no substrate for integration. Relevant to all delivery options is the risk for the potential introduction of mutations at off-target sites due to binding and cleavage activity of editors at sites with sequence similarity to the actual target site (Graham et al., 2009; Fu et al., 2013; Kim et al., 2014; Young et al., 2020). Plasmid-encoded editors have a longer activity window, which might increase the off-target risk compared to editors delivered as RNA or RNP. With well-designed editors and in the context of livestock, the potential risk for introducing off-target mutations appears to be relatively low even when editors were delivered by plasmid (Akcakaya et al., 2018; Wang et al., 2018b; Li et al., 2018; Jivanji et al., 2021). Comparing the efficiency of introducing HDR edits dependent on the delivery of gRNA/Cas9 editors as DNA, RNA or RNP across two genes and three different target sites showed some degree of variability for generating mosaic embryos comprising HDR-edited alleles (Table 4). By contrast, there was no difference in the efficiency to generate embryos fully converted into the HDR-edited genotype. This was different for the introduction of the PMEL mutation with TALENs TL17/TR217. Here, delivery as plasmid resulted in a 13-fold higher percentage of fully converted embryos compared to the RNA delivery (Table 4). This would strongly argue for plasmid delivery in this particular case, if full conversion has priority and the risk of potential integration can be tolerated for the intended application of producing edited animals.

Microinjection is considered to be a minor manipulation of the embryo and the injection of an inert dye was previously reported to not adversely impact on the *in vitro* development of bovine embryos (Bogliotti et al., 2016). In our hands, the injection of editing molecules reduced the total development to the blastocyst stage from 50% for non-injected controls to 31–36% for injected embryos (Table 6). This was consistent with the impacts from injection of editing tools being observed by others (Hennig et al., 2020). By contrast, the development to blastocysts of transferable quality (G1-2) was mainly unaffected (13% vs 13–22%, Table 6) and was only reduced for injection of gRNA 632, Cas9 mRNA (6%). Furthermore, biopsied embryos were not compromised by cryopreservation with an almost 100% recovery rate, independent of their injection status.

An earlier study using biopsies as screening tool for edited sheep embryos found a relative low correlation between biopsies and fetuses produced from the embryos (Vilarino et al., 2018). The screen used endpoint PCR to detect differently sized fragments of the amplified target region edited for the introduction of a deletion to knockout PDX1 while, what was considered the actual genotype, was determined by a ddPCR assay. Our biopsy screens were based on quantitative ddPCR assays with hybridization probes for the HDR edits to determine the contribution of HDR alleles vs all other alleles in individual embryos. In a previous study, we could confirm the predictive value of the biopsies by comparative next generation sequencing and ddPCR showing good correlation between biopsy and resulting calves (Wei et al., 2018). This suggests that biopsy screening might be a suitable strategy to evaluate the editing success prior to the production of live animals. Some edited embryos that were biopsied and cryopreserved have now been transferred to recipients for development to term which can be expected to provide additional information on the accuracy of predicting complete conversion into precision edited genotypes from biopsy samples.

In summary, we showed the ability to readily convert bovine embryos into non-mosaic, precisely HDR-edited genotypes for several naturally occurring sequence variants in two genes. The best conversion rate (48%) was achieved with plasmid delivery and TALEN as editors versus 11% as the highest conversion rate with gRNA/Cas9 editors when delivered as RNA. Plasmid delivery is commonly not favoured due to the additional risk of integration but should not be overlooked if a greater efficiency might justify the increased risk profile. Although gRNA/Cas9 editors dominate due to the ease of use, the ‘older’ editing platforms ZFNs and TALENs should not be discounted and might offer benefits resulting from the functional differences between the editing platforms. Our study further showed that the injection of editing tools had no effect on the production of transferable quality blastocyst stage embryos and that biopsying embryos was compatible with cryopreservation. This lends support for biopsies as a suitable screening strategy for fully converted embryos with the potential to integrate it with embryonic genomic selection procedures. Together with the rapidly improving editing tools, such as enhanced prime editors, the approach provides an exciting outlook for the efficient introgression of natural variants into elite livestock by genome editing in the future.

## Data Availability

The original contributions presented in the study are included in the article/Supplementary Material, further inquiries can be directed to the corresponding author.
