# Supplementary material for: Cytoplasmic Injection of Zygotes to Genome Edit Naturally Occurring Sequence Variants Into Bovine Embryos
Source: Front Genet. 2022 Jul 11;13:925913. doi: 10.3389/fgene.2022.925913 (PMC9310181; doi:10.3389/fgene.2022.925913)
Supplement: Supplementary file 1 [file DataSheet2.pdf]

**PRLR, wt**		AGA	TAT	TCT	TAC	CAC	AAC	ATT	GCT	GAC	GTG	TG**T**	GAG	CTG	GCC	
	430	R	Y	S	Y	H	N	I	A	D	V	C	E	L	A	443
**PRLR, p.C440***		AGA	TAT	TCT	TAC	CAC	AAC	ATT	GCT	GAC	GTG	TG**A**	GAG	CTG	GCC	
	430	R	Y	S	Y	H	N	I	A	D	V	*				440
**PRLR, wt**		CAA	ACA	GAC	CAA	CAT	GCT	TTA	AAA	GCC	T**C**A	AAA	ACC	ATT	GAA	
	456	Q	T	D	Q	H	**A**	L	K	A	S	K	T	I	E	456
**PRLR, p.L462***		CAA	ACA	GAC	CAA	CAT	GTT	TAA	AAG	CCT	CAA	AAA	CCA	TTG	AAA	
	456	Q	T	D	Q	H	**V**	*								456
**PRLR, p.S465***		CAA	ACA	GAC	CAA	CAT	GCT	TTA	AAA	GCC	T**A**A	AAA	ACC	ATT	GAA	
	456	Q	T	D	Q	H	A	L	K	A	*					465

Supplementary Figure 2. Sequence of wt and HDR-edited alleles. Nucleotide and amino acid sequences of relevant regions are shown for the wt allele and alleles with edited p.C440*, p.L462* and p.S465* mutations. Numbers refer to amino acid sequence positions in *PRLR*. Gene sequence changes are indicated in bold, the nucleotide deleted in the p.L462* edited allele is underlined. Premature stop codons and the amino acid change (A to V in pL462*) are in bold.
