# Supplementary material for: Cytoplasmic Injection of Zygotes to Genome Edit Naturally Occurring Sequence Variants Into Bovine Embryos
Source: Front Genet. 2022 Jul 11;13:925913. doi: 10.3389/fgene.2022.925913 (PMC9310181; doi:10.3389/fgene.2022.925913)
Supplement: Supplementary file 2 [file DataSheet1.pdf]

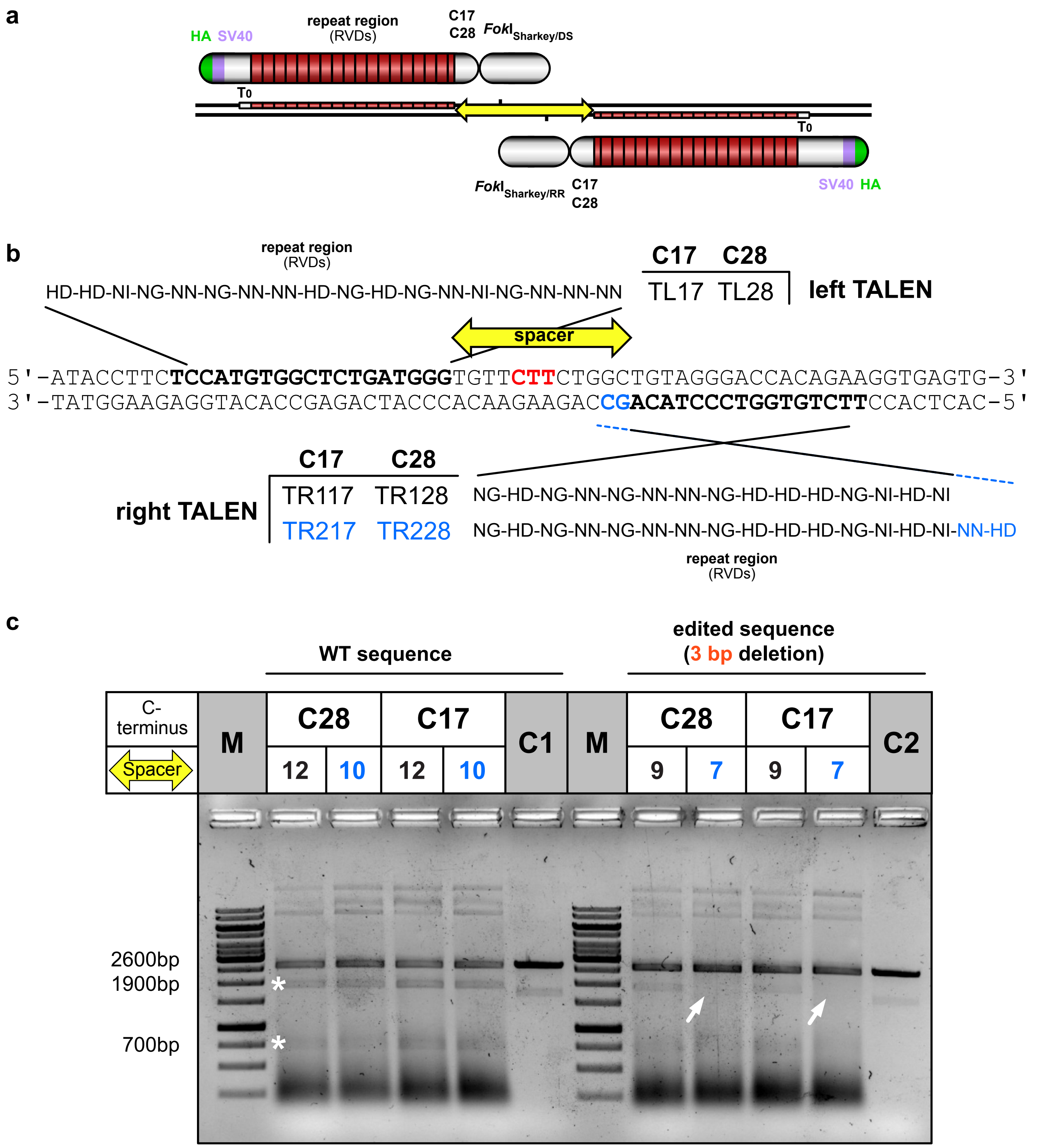

Supplementary Figure 1. Design and *in vitro* activity of TALEN pairs. **(a)** TALEN design. TALEN were constructed with N-terminal HA-tag (green) and SV40 NLS (purple), either 17 aa (C17) or 28 aa (C28) C-terminal domain, and heterodimerizing Sharkey/DS and Sharkey/RR FokI domains. **(b)** TALEN binding sites. Target DNA sequence (bold) and matching RVDs of left and right TALEN, respectively. Two of the right TALEN are two RVDs longer (blue), resulting in a shorter spacer region (yellow). The edited sequence contains a three bp deletion (red). **(c)** *in vitro* cleavage assay of wild type and edited sequence using different TALEN pairs. Combinations of left and right TALENs were *in vitro* transcribed and translated and incubated with linearized plasmid containing either subcloned wild type (WT, C1) or edited sequence (C2). Restriction fragments (white stars) show TALEN activity. The TALEN had either C17 or C28 C-termini. Combinations including the blue right TALEN did not show activity on the edited sequence (white arrows) and were thus able to efficiently discriminate between WT (10 bp spacer) and edited sequence (7 bp spacer).
